# Gut Microbiota as Mediator and Moderator Between Hepatitis B Virus and Hepatocellular Carcinoma: A Prospective Study

**DOI:** 10.1002/cam4.70454

**Published:** 2024-12-19

**Authors:** Bingren Hu, Yi Yang, Jiangqiao Yao, Ganglian Lin, Qikuan He, Zhiyuan Bo, Zhewei Zhang, Anlvna Li, Yi Wang, Gang Chen, Yunfeng Shan

**Affiliations:** ^1^ Department of Hepatobiliary and Pancreatic Surgery The First Affiliated Hospital of Wenzhou Medical University Wenzhou China; ^2^ Key Laboratory of Diagnosis and Treatment of Severe Hepato‐Pancreatic Diseases of Zhejiang Province The First Affiliated Hospital of Wenzhou Medical University Wenzhou Zhejiang China; ^3^ Department of Epidemiology and Biostatistics, School of Public Health Wenzhou Medical University Wenzhou China; ^4^ The First Clinical College Wenzhou Medical University Wenzhou China; ^5^ Zhejiang‐Germany Interdisciplinary Joint Laboratory of Hepatobiliary‐Pancreatic Tumor and Bioengineering The First Affiliated Hospital of Wenzhou Medical University Wenzhou Zhejiang China

**Keywords:** gut‐liver axis, gut microbiota, hepatitis B virus, hepatocellular carcinoma, machine learning

## Abstract

**Background:**

The impact of gut microbiome on hepatitis B virus (HBV)‐related hepatocellular carcinoma (HCC) is unclear. We aimed to evaluate the potential correlation between gut microbiome and HBV‐related HCC and introduced novel machine learning (ML) signatures based on gut microbe to predict the risk of HCC.

**Materials and Methods:**

A total of 640 patients with chronic liver diseases or HCC were prospectively recruited between 2019 and 2022. Fecal samples were collected and subjected to 16S rRNA gene sequencing. Univariate and multivariate logistic regression was applied to identify risk characteristics. Several ML methods were employed to construct gut microbe‐based models and the predictive performance was evaluated.

**Results:**

A total of 571 patients were involved in the study, including 374 patients with HCC and 197 patients with chronic liver diseases. After the propensity score matching method, 147 pairs of participants were enrolled in the analysis. *Bacteroidia* and *Bacteroidales* were demonstrated to exert mediating effects between HBV and HCC, and the moderating effects varied across *Bacilli*, *Lactobacillales*, *Erysipelotrichaceae*, *Actinomyces*, and *Roseburia*. HBV, alpha‐fetoprotein, alanine transaminase, triglyceride, and Child‐Pugh were identified as independent risk factors for HCC occurrence. Seven ML‐based HBV‐gut microbe models were established to predict HCC, with AUCs ranging from 0.821 to 0.898 in the training set and 0.813–0.885 in the validation set. Furthermore, the merged clinical‐HBV‐gut microbe models exhibited a comparable performance to HBV‐gut microbe models.

**Conclusions:**

Gut microbes are important factors between HBV and HCC through its potential mediating and moderating effects, which can be used as valuable biomarkers for the pathogenesis of HBV‐related HCC.

**Abbreviations**
AFP: alpha‐fetoprotein
AI: artificial intelligence.
ALT: alanine transaminase
BMI: body mass index
CART: classification and regression tree
CI: confidence interval
DBIL: direct bilirubin
DCA: decision curve analysis
GBM: gradient boosting machine
HBV: hepatitis B virus
HCC: hepatocellular carcinoma
HCV: hepatitis C virus
LEfSe: linear discriminant analysis effect size
LR: logistic regression
ML: machine learning
NCCN: National Comprehensive Cancer Network
NN: neural network
NNM: nearest neighbor matching
OR: odds ratio
PCoA: principal coordinates analysis
PLC: primary liver cancer
PSM: propensity score matching
PT: prothrombin time
RF: random forest
ROC: receiver operating characteristic
SD: standard deviation
SE: standard error
SVM: support vector machine
TBIL: total bilirubin
TG: triglyceride
XGBoost: eXtreme gradient boosting

## 1 Introduction

Primary liver cancer (PLC), including hepatocellular carcinoma (HCC) (85% of cases), ranks as the third leading cause of cancer‐related mortality with over 800,000 deaths worldwide in 2020 [1]. Epidemiologically, it has complex risk factors that comprise aflatoxin, alcohol, hereditary metabolic liver disease, and hepatitis virus infection [2], especially hepatitis B virus (HBV), which contributes maximum to HCC in the Asia‐Pacific region. Moreover, the pathogenic aspects of HBV‐induced HCC differ depending on a variety of factors, influencing the clinical outcomes. Therefore, it is imperative to identify biological mediators underneath the HBV–HCC interaction, with an emphasis on developing surveillance strategies and improving individual management.

Emerging evidence has demonstrated an intrinsic linkage between the gut microbiome and HBV‐induced liver diseases for its cross‐talk with the gastrointestinal tract, termed the “gut‐liver” axis [3, 4]. Indeed, significant changes in gut microbial composition were determined in HBV patients characterized by a gain in pathogenic taxa including *Firmicutes*, *Prevotella*, *Proteobacteria*, and *Streptococcus* [5, 6]. Simultaneously, HBV has been shown to contribute to microbial translocation over the impaired intestinal barrier, resulting in an overactive long‐term immune response that is implicated in the progression of HCC [7]. The study by Liu et al. depicted the alterations in gut microbiota between HCC patients with HBV or without HBV/hepatitis C virus (HCV) infection [8]. Moreover, the altered gut bacterial composition, or dysbiosis, could in turn regulate the HBV‐related hepatocarcinogenesis via diverse mechanisms, such as microorganism–host genetic modification, microbiota‐derived metabolites (e.g., short‐chain fatty acid, bile acid, and choline) [9, 10], immune microenvironment regulation and inflammation‐related signaling (Toll‐like receptor 4 pathway) [11]. In view of the uniquely bidirectional communication among gut microbes and HBV‐induced HCC, most of those previous studies merely focused on the variations of microbial composition, while a comprehensive analysis of the intertwined effect along the progression from HBV infection to HCC onset remains to be clarified.

Recently, the data‐driven machine learning (ML) technique has been broadly employed in many areas of cancer biology with its exceptional accuracy compared to the experimentally determined structures and has also successfully tackled the challenge of disease prediction and prognosis [12]. Accordingly, Wei et al. [13] innovatively proposed an optimized early warning system for lung cancer risk using a cutting‐edge ML method named XGBoost, with favorable reliability and robustness. The multi‐labeled ML classifier was further applied to predict whether the cancer type is primary or metastatic type based on the CpG methylation level [14]. Concurrently, our previous studies have verified the practical value of ML algorithm in the field of PLC management, including the recurrence of intrahepatic cholangiocarcinoma [15] and the monitoring of Lenvatinib response in HCC [16]. In addition, the ML approach may also exhibit overlapping gut microbial signatures in that the non‐invasive microbiome‐based multi‐class model achieved impressive performance for disease classification, which complemented the cancer diagnostics [17]. For this reason, the so‐called ML‐featured microbiome taxonomic profiles might be a promising tool in clarifying the evolution from HBV to HCC occurrence. Here, we prospectively recruited a group of individuals with chronic liver diseases or HCC and analyzed the differential taxa as mediators or regulators. We disclosed a potential involvement of gut microbiota in HBV‐induced HCC pathogenesis and constructed valuable ML models based on the significant gut microbiota to predict the occurrence of HCC.

## 2 Materials and Methods

### 2.1 Patient Enrollment

**FIGURE 1:**
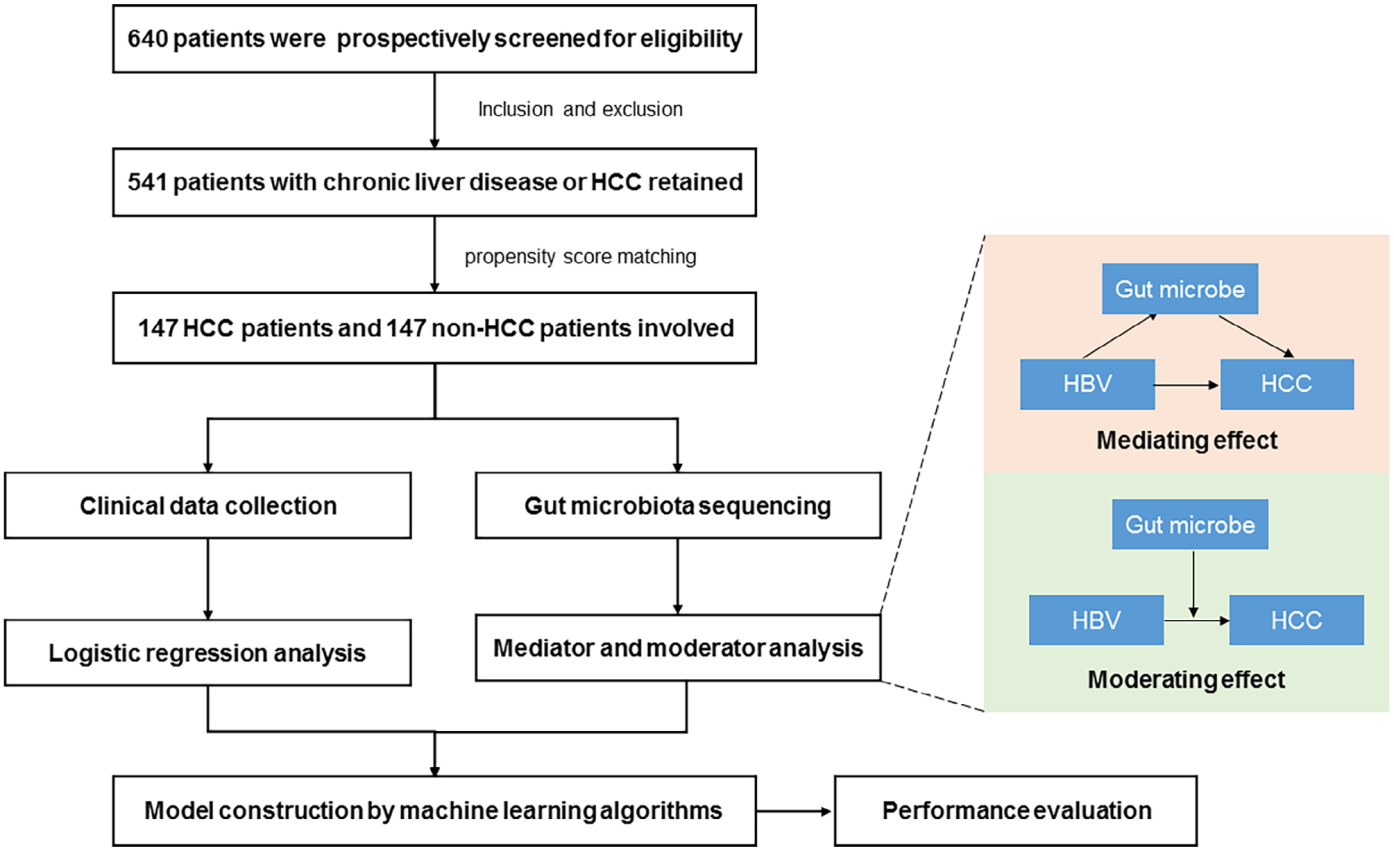
Flowchart of this study.

Patients with chronic liver diseases or HCC were prospectively recruited between June 2019 and December 2022 from the First Affiliated Hospital of Wenzhou Medical University. HCC was clinically diagnosed via the National Comprehensive Cancer Network (NCCN) standards [18]. Key inclusion criteria were as follows: (i) patients between 18 and 80 years old; (ii) no exposure to antibiotics or probiotics within 8 weeks before sampling; and (iii) consent to sign informed consent. Key exclusion criteria were as follows: (i) combined with other malignant tumors; (ii) unqualified stool samples for analysis; (iii) incomplete clinical data; and (iv) combined with autoimmune disorders, metabolic diseases, or other viral hepatitis. Relevant clinical data such as age, gender, body mass index (BMI), diet, smoking history, alcohol drinking history, liver cirrhosis, and serum variables were collected. Figure 1 shows the flowchart of the study.

This study was approved by the Ethics Committee in Clinical Research of the First Affiliated Hospital of Wenzhou Medical University (KY2023‐R256) and adhered to the Declaration of Helsinki. Written informed consent was obtained from each patient before the research.

### 2.2 16S rRNA Gene Sequencing and Analysis

All stool samples were freshly collected and frozen at −80°C within half an hour after sampling. The EZNA Stool DNA Kit (D4015, Omega Inc., USA) was used to extract the fecal sample's genomic DNA. The 341F (5′‐CCTACGGGNGGCWGCAG‐3′) and 805R (5′‐GACTACHVGGGTATCTAATCC‐3′) were utilized as the primers. To identify and rectify the original readings, Divisive Amplicon Denoising Algorithm 2 was chosen in this study. The distribution differences of the gut microbiota in the two groups were discovered via the principal coordinated analysis (PCoA). To filter the discriminating taxa across the two groups, the linear discriminant analysis effect size (LEfSe) was utilized with the criteria of LDA score > 2 (Wilcoxon < 0.05). Furthermore, the Phylogenetic Investigation of Communities by Reconstruction of Unobserved States 2 (PICRUSt2) method was employed to delve deeper into the specific biological functional differences arising from variations in the gut microbiota between the two groups [19].

### 2.3 Mediation/Regulation Effect

The mediation/regulation effect was employed to explore the role of intestinal flora in the relationship between HBV and HCC using Bootstrap [20] and Johnson–Neyman [21] techniques. The occurrence of HCC was utilized as the dependent variable, HBV conditions of the groups were taken as the independent variables, and the different intestinal microorganisms of the two groups were used as the mediation/regulation variables. The intermediary effect was characterized by the 95% confidence interval (CI) excluding 0, and the regulation effect was defined as an odds ratio (OR) with a *p* value < 0.05 considered significant.

### 2.4 Machine Learning to Construct Gut Microbiota‐Based Models

The “Scikit‐learn 0.24.0” python package was used to perform the ML analysis. Seven excellent ML classifiers were employed to construct HBV‐Intestinal flora axis models based on the gut microbes which had the mediation/regulation effects between HBV and HCC, including Classification and Regression Tree (CART), Gradient Boosting Machine (GBM), Logistic Regression (LR), Neural Network (NN), Random Forest (RF), Support Vector Machine (SVM), and eXtreme Gradient Boosting (XGBoost). Patients were randomly assigned as training and validation set at a ratio of 7:3. To increase the generalization of the results, we further implemented repeated 10‐fold cross‐validation (CV) of the training dataset to avoid overfitting when training the ML classifiers. Grid search methods were utilized to adjust parameters to improve the performance of seven ML models. To address the issue of redundancy among variables, we employed the Least Absolute Shrinkage and Selection Operator (LASSO) regression analysis to screen significant gut microbes and mitigate the risk of collinearity. Receiver operating characteristic (ROC) curves were employed to evaluate the performance of various ML models. The calibration curve analysis and decision curve analysis (DCA) were utilized to test the robustness and clinical applicability of the seven ML models [22].

### 2.5 Statistical Analysis

All statistical analyses were performed with R software (Version 4.2.2) and Python software (Version 3.9.R). Continuous data were analyzed by *t*‐test or Mann–Whitney *U* test, shown as mean ± standard deviation (SD) or medians (interquartile range) according to the distribution. Univariate and multivariate logistic regression analyses were performed to identify clinical risk factors. Multivariable logistic regression analyses were carried out on variables with a *p* value < 0.1 in the univariate analysis. The propensity score matching (PSM) method was applied through the “MatchIt” R package, to adjust the confounding factors such as age, gender, smoking, alcohol drinking history, and liver cirrhosis. *Z*‐score standardization was used to normalize clinical data and microbial data to eliminate the dimensional influence between different types of features, so as to facilitate subsequent model development. The nearest neighbor matching method (NNM) was utilized with the caliper value of 0.11. The “mediation” and “interactions” R packages were hired for the mediation/regulation effect analyses. *p* value < 0.05 was deemed statistically significant.

## 3 Results

### 3.1 Baseline Characteristics of Patients

A total of 640 patients with chronic liver diseases or HCC were initially registered. Following the inclusion and exclusion criteria, 197 controls and 374 cases were enrolled in the study, and 147 pairs of participants were eventually included in the analysis after the PSM. The baseline characteristics of patients before and after PSM were displayed in Table S1. Univariate and multivariate logistic regression was utilized to determine significant clinical factors that are associated with HCC occurrence. As shown in Table S2, HBV infection (*p* < 0.001), alpha‐fetoprotein (AFP, *p* < 0.001), total bilirubin (TBIL, *p* = 0.023), direct bilirubin (DBIL, *p* = 0.002), alanine transaminase (ALT, *p* = 0.003), triglyceride (TG, *p* < 0.001), Child‐Pugh (*p* = 0.001), and prothrombin time (PT, *p* = 0.042) were associated with HCC occurrence through the univariate regression analysis. The variables with *p* value < 0.1 were then included in multivariate regression analysis with the “Forward LR” algorithm. HBV infection (*p* < 0.001), AFP (*p* < 0.001), ALT (*p* = 0.038), TG (*p* = 0.005), and Child‐Pugh (*p* = 0.011) were identified as independent risk factors for the hepatocarcinogenesis.

### 3.2 Microbial Difference Analysis by 16S rRNA Gene Sequencing

**FIGURE 2:**
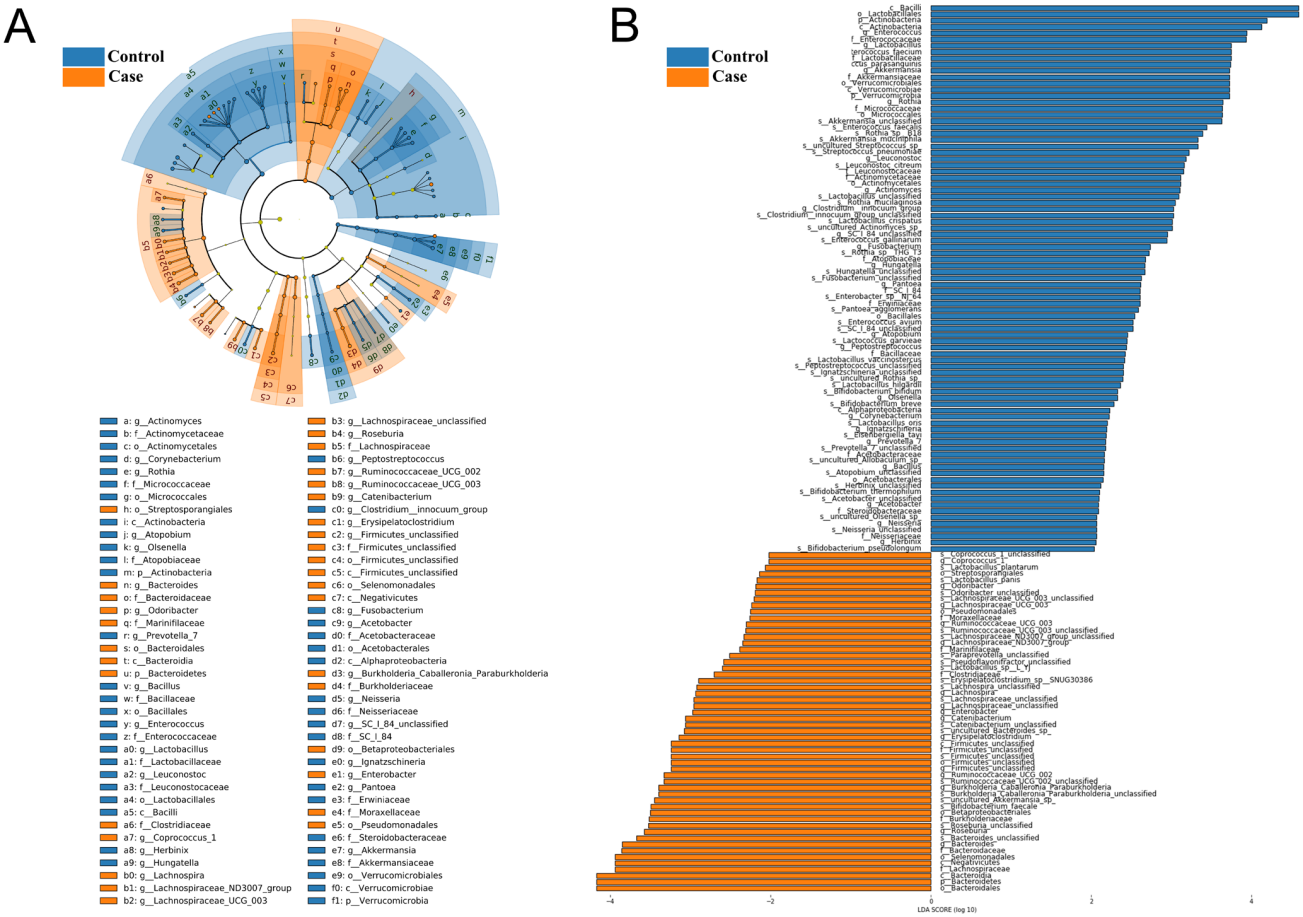
Differential microbial taxa between the control and HCC groups. (A) Differences of predominant taxa between patients with HCC and patients with chronic liver diseases in a phylogenetic tree with cladogram calculated by linear discriminant analysis effect size (LEfSe) algorithm with the LDA score > 2 (Wilcoxon < 0.05). (B) Predominant microbial taxa between the two groups were shown in the bar plot (Wilcoxon < 0.05, LDA > 2). HCC, hepatocellular carcinoma; HBV, hepatitis B virus; LEfSe, linear discriminant analysis effect size; LDA, linear discriminant analysis.

As critical parameters, alpha and beta diversity were utilized for gut microbiota analysis. Results in Figure S1 illustrated that the HCC group showed markedly increased α‐diversity versus the control group indicated by the Shannon (*p* = 0.034) and Simpson (*p* = 0.018) indices. To annotate the distributional difference, the weighted Unifrac PCoA was used to calculate the β‐diversity between the two groups. The HCC group exhibited statistically separated clustering from the control group (*p* = 0.001). Furthermore, the LEfSe algorithm was used to estimate the taxonomic difference with the criteria of LDA score > 2, and the differential microbial taxa between the control and HCC groups are shown in Figure 2. In short, the gut microbial profiles alter in HCC patients and differ from those of other conditions. Moreover, the results of the PICRUSt2 analysis at Kyoto Encyclopedia of Genes and Genomes (KEGG) pathway revealed significant differences in various metabolic pathways between the two groups, such as porphyrin and chlorophyll metabolism (*p* = 0.032), glycine, serine and threonine metabolism (*p* = 0.029), bacterial chemotaxis (*p* = 0.031), apoptosis (*p* = 0.027), and arginine and proline metabolism (*p* = 0.0004) (Figure S2).

### 3.3 Intermediary and Regulatory Effect Analysis of Gut Microbiome

The Bootstrap and Johnson–Neyman methods were followed to examine whether the gut microbial taxa were potential mediator or moderator variables that accounted for the connection between HBV infection and HCC incidence. Accordingly, *Bacteroidia* at the class level (*p* = 0.042, 95% CI: [0.0002, 0.0400]) and *Bacteroidales* at the order level (*p* = 0.036, 95% CI: [0.0003, 0.0400]) were employed as intermediary factors between HBV and HCC that made up 2.73% and 2.74% of the total effects, respectively, and the direct effects showed statistical significance as well (Table 1). Subsequently, Table 2 presented the regulatory effects of gut microbes at different levels between the two groups, with HBV infection as the independent variable. Results showed that the *Bacilli* (*p* = 0.028, OR = 0.976, 95% CI: [−0.046, −0.003]), *Lactobacillales* (*p* = 0.030, OR = 0.976, 95% CI: [−0.046, −0.002]), and *Actinomyces* (*p* = 0.047, OR = 0.066, 95% CI: [−5.392, −0.035]) exhibited negative regulatory effects, whereas the *Erysipelotrichaceae* (*p* = 0.014, OR = 1.296, 95% CI: [0.053, 0.466]), *Roseburia* (*p* = 0.017, OR = 1.887, 95% CI: [0.111, 1.158]), and *Roseburia_Unclassified* (*p* = 0.039, OR = 2.022, 95% CI: [0.035, 1.373]) displayed positive regulatory effect that promoted the development of HBV‐related HCC. Taken together, these data reflected the mediating and moderating involvement of gut microbiota during HBV‐related hepatic carcinogenesis.

**TABLE 1:** The mediating effects of intestinal microflora at all levels between hepatitis B and hepatocellular carcinoma.

Level	Intestinal flora	Effect	95% CI	*p*	Proportion of mediating effect
Class	*Bacteroidia*	Mediating effect	[0.0002, 0.0400]	0.042	2.73%
		Direct effect	[0.3979, 0.5800]	0.042	
		Total effect	[0.4138, 0.6000]	< 0.001	
Order	*Bacteroidales*	Mediating effect	[0.0003, 0.0400]	0.036	2.74%
		Direct effect	[0.3922, 0.5900]	0.036	
		Total effect	[0.4110, 0.6100]	< 0.001	

Abbreviation: CI, confidence interval.

**TABLE 2:** The moderating effects of intestinal microflora at all levels between hepatitis B and hepatocellular carcinoma.

Level	Intestinal flora	Effect	SE	OR	95% CI	*p*
Class	*Bacilli*	Main effect	0.281	9.463	[1.696, 2.798]	< 0.001
		Regulatory effect	0.011	0.976	[−0.046, −0.003]	0.028
Order	*Lactobacillales*	Main effect	0.281	0.459	[1.696, 2.798]	< 0.001
		Regulatory effect	0.011	0.976	[−0.046, −0.002]	0.030
Family	*Erysipelotrichaceae*	Main effect	0.309	11.458	[1.833, 3.044]	< 0.001
		Regulatory effect	0.105	1.296	[0.053, 0.466]	0.014
Genus	*Actinomyces*	Main effect	0.302	8.321	[1.528, 2.710]	< 0.001
		Regulatory effect	1.367	0.066	[−5.392, −0.035]	0.047
	*Roseburia*	Main effect	0.320	12.379	[1.888, 3.144]	< 0.001
		Regulatory effect	0.267	1.887	[0.111, 1.158]	0.017
Species	*Roseburia_Unclassified*	Main effect	0.316	11.888	[1.856, 3.095]	< 0.001
		Regulatory effect	0.342	2.022	[0.035, 1.373]	0.039

Abbreviations: CI, confidence interval; OR, odds ratio; SE, standard error.

### 3.4 Construction of Gut Microbiome‐Based Models by Machine Learning Methods

**FIGURE 3:**
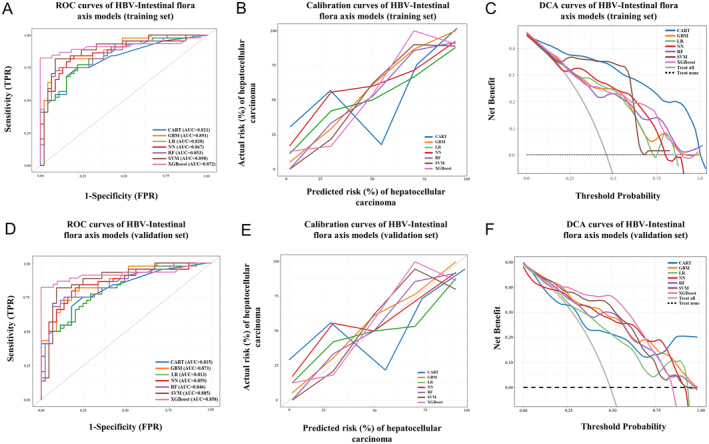
Construction of gut microbe‐based models in predicting HCC through seven ML algorithms. (A) ROC curves of seven ML gut microbe‐based models in the training set. (B) Calibration plots of seven ML gut microbe‐based models in the training set. (C) Decision curve analysis of seven ML gut microbe‐based models in the training set. (D) ROC curves of seven ML gut microbe‐based models in the validation set. (E) Calibration plots of seven ML gut microbe‐based models in the validation set. (F) Decision curve analysis of seven ML gut microbe‐based models in the validation set. CART, classification and regression tree; GBM, gradient boosting machine; HBV, hepatitis B virus; HCC, hepatocellular carcinoma; LR, Logistic Regression; ML, machine learning; NN, neural network; RF, random forest; ROC, receiver operating characteristic curve; SVM, support vector machine; TPR, true positive rate; FPR, false positive rate; XGBoost, eXtreme gradient boosting.

**FIGURE 4:**
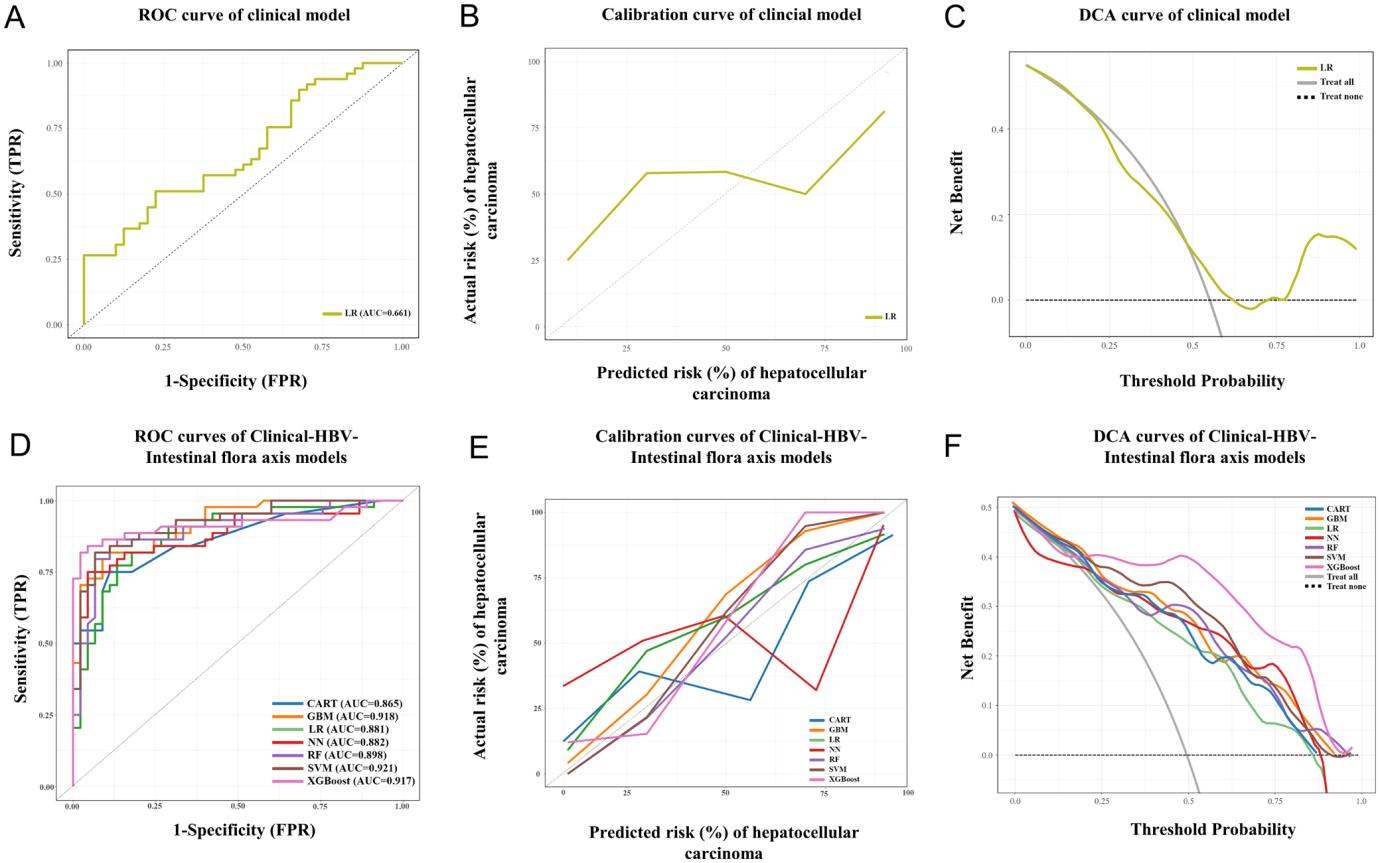
Construction of clinical models and clinical‐HBV‐intestinal flora axis models in predicting HCC through seven ML algorithms. (A) ROC curve of the clinical model. (B) Calibration plot of the clinical model. (C) Decision curve analysis of the clinical model. (D) ROC curves of the clinical‐HBV‐intestinal flora axis models. (E) Calibration plots of clinical‐HBV‐intestinal flora axis models. (F) Calibration plots of clinical‐HBV‐intestinal flora axis models. CART, classification and regression tree; FPR, false positive rate; GBM, gradient boosting machine; HBV, hepatitis B virus; HCC, hepatocellular carcinoma; LR, logistic regression; ML, machine learning; NN, neural network; RF, random forest; ROC, receiver operating characteristic curve; SVM, support vector machine; TPR, true positive rate; XGBoost, eXtreme gradient boosting.

Having recognized the presence of a microbial signal as a mediator and moderator effect on HCC at the coarse stage of general community composition. ML method was further integrated to construct HBV‐gut microbe axis‐based models to predict HCC incidence. According to the results of the multivariable analysis, we established a clinical feature‐based model by logistic regression method with an AUC of 0.661 (Figure 4A). The calibration curve and DCA showed an unsatisfactory performance (Figure 4B,C). According to the results of LASSO regression analysis (Figure S3), several microbial features were incorporated to develop ML modes, including Class_Bacteroidia, Order_Bacteroidales, Order_Lactobacillales, Family_Erysipelotrichaceae, Genus_Actinomyces, Genus_Roseburia, and Species_Roseburia_Unclassified. After that, seven ML gut microbe‐based models were developed in the training set, with AUCs ranging from 0.821 to 0.898 (Figure 3A). The results of the 10‐fold CV showed that the GBM algorithm achieved the highest AUC of 0.879 ± 0.12, while the other six ML algorithms all achieved an average AUC > 0.8. In the validation set, the AUCs were relatively stable for predicting HCC and were consistent with the performance of the training set, ranging from 0.813 to 0.885 (Figure 3D). The calibration curves and DCA also showed a favorable performance (Figure 3E,F). Collectively, the clinical features and microbial features were integrated to form combination models, known as the clinical‐HBV‐intestinal flora axis models. The predictive performance was comparable to the HBV‐gut flora‐based models, with AUCs from 0.865 to 0.921 (Figure 4D). Consistently, the calibration curves and DCA also confirmed the satisfactory performance of the combination models (Figure 4E,F).

## 4 Discussion

As a silent malignancy, HBV‐induced HCC remains a formidable problem that requires substantial efforts to mitigate its adverse burden. In the present study, we provided comprehensive evidence that the variations in the gut microbial composition appeared to be powerful mediators or regulators in HBV‐related HCC. Notably, it was the first time that we attempted to apply the microbial counterparts to promote ML classifiers for HCC prediction with impressive accuracy. We recommend that the ML‐derived HBV‐gut microbe axis could be a promising, noninvasive, and bench‐to‐bedside tool for anticipating the occurrence of HCC.

Given the dismal survival of HCC, there has been a surge of interest in determining clinical biomarkers for evaluating the prognosis [23, 24]. Accordingly, several optimal candidates have been reported, including serum markers, tumor burden, vascular invasion, and extrahepatic spread [25, 26]. In addition, a landmark work reported by Lu and his colleagues [27] innovatively defined three subgroups of HCC based on the prominent risk factor, HBV infection, which corresponded with prognostic survival characteristics. Consistently, our study found that HBV, AFP, ALT, triglyceride, and Child‐Pugh class were independent risk factors for HCC. Nevertheless, the prediction of HCC with the clinical markers was far away from satisfaction (AUC of 0.661), and a more robust biomarker‐derived classifier was needed.

Anecdotal evidence has revealed an inherent link between PLC and gut microbiome [28, 29] using the taxonomic resolution of 16S rRNA amplicons, notably including HCC with HBV infection [4]. In particular, the gut microbiota has also been identified to reliably predict HCC incidence with cross‐regional validation [30], highlighting its noninvasive potential for diagnostic categorization. In this prospective cohort study, we delineated the bacterial composition of fecal samples with 16S rRNA sequencing in patients with HCC and chronic liver diseases. The taxa heterogeneity and distribution indicated by β‐diversity and the LEfSe analysis distinctly separated HCC from the control group. Of note, the profound microbe‐induced impact was subsequently investigated as the HBV‐related HCC progressed, and this was the emphasis of our work. As a result, several microbial taxa were proven as decisive mediators and regulators between HBV and HCC, such as *Bacteroidia*, *Bacteroidales*, *Bacilli*, *Lactobacillales*, *Erysipelotrichaceae*, *Actinomyces*, and *Roseburia*, some of which had been confirmed to be involved in the hepatocarcinogenesis. Coincidentally, microbiota dysbiosis appeared in the HCV infection, with continuously dysregulated levels of the class *Bacilli* and the order *Lactobacillales* as a consequence of the HCV‐related HCC [31]. In a preclinical trial, *Erysipelotrichaceae* [32] was recognized to correspond with the changing patterns of the gut microbiome in spontaneous female *LTsc1KO* mice model and might act as a valid target for future HCC diagnosis and prevention. In addition, Zhang et al. [33] made one important step in the cross‐talk of microbial dysbiosis and chronic HBV patients, which was accompanied by a substantial alteration in the genus *Actinomyces* for a novel therapeutic perspective. In line with that, our data provided an empirical reference that the microbial feature either mediated the relationship between HBV and HCC or was a moderator in the progression of HBV‐induced HCC, underscoring the significance of particular microbial taxa in screening individuals with HBV who would ultimately develop HCC with certain convincing bioinformatics techniques.

Currently, a breakthrough in the field of ML‐related strategy has inspired the implementation of artificial intelligence (AI) for HCC pre‐screening, diagnosis, and management [34, 35, 36, 37]. In our study, to make the results more compelling, seven ML algorithms were applied to construct gut microbe‐based models, which all achieved favorable performance with stringent internal validation, indicating the satisfactory clinical value of ML microbiome in predicting the incidence of HCC. Apart from that, several clinical indicators were demonstrated to be related to the commensal community of intestinal microbiota, including AFP [38], ALT, and TG [39]. Consequently, by inputting those clinical features with statistical coefficients into our ML algorithms, seven comparable clinical‐HBV‐intestinal microbe signatures were evaluated for the probability of HCC. For HBV patients who were prone to high risks, appropriate tactics should be put on the agenda to postpone or even prevent the disease deterioration, which was promising to translate into practical application and to provide timely recommendations for clinicians. The ML‐driven microbial analysis can be used as a surrogate technique for the prediction of HBV‐related HCC.

In spite of the inspiring results, there are still certain limitations to our study. The data were obtained from one single center, further study was needed to verify the generalizability and reproducibility of the results. Sequential samples were derived from one specific disease status without dynamic fecal samples of the same individual over time, it would be more persuasive to outline the comprehensive landscape of taxonomic profiling during the deterioration. Furthermore, the potential mechanism underlying the biological function of the gut microbiome in HBV‐related HCC remains ambiguous, and it was innovative to recapitulate the HCC microbiota with animal models. Lastly, although clinical features can provide some predictive potential for HCC, the predictive performance was inferior based on the current data. This may be partly due to the limited sample size and heterogeneity of different studies, and further studies based on larger cohorts are still warranted to validate the results.

## 5 Conclusion

The present research characterized the mediating and regulating functions of the gut microbiome in the relationship between HBV and HCC, which were then incorporated to build distinct ML‐based microbial signatures that enabled robust performance for HBV‐induced HCC identification. Beyond the potential for diagnosis, we presume that the panel of microbial taxa may be relevant to HBV‐related hepatocarcinogenesis, providing an alternative insight into proper HCC intervention.

## Author Contributions

**Bingren Hu:** conceptualization (equal), writing – original draft (equal). **Yi Yang:** formal analysis (lead), methodology (lead). **Jiangqiao Yao:** data curation (equal), resources (equal). **Ganglian Lin:** data curation (equal), resources (equal). **Qikuan He:** investigation (equal), resources (equal). **Zhiyuan Bo:** conceptualization (equal), resources (equal), writing – original draft (equal). **Zhewei Zhang:** resources (equal). **Anlvna Li:** resources (equal). **Yi Wang:** supervision (equal), writing – review and editing (equal). **Gang Chen:** funding acquisition (equal), supervision (equal), writing – review and editing (equal). **Yunfeng Shan:** conceptualization (equal), supervision (equal), writing – review and editing (equal).

## Ethics Statement

This study was approved by the Ethics Committee in Clinical Research of the First Affiliated Hospital of Wenzhou Medical University (KY2023‐R256) and adhered to the Declaration of Helsinki.

## Consent

Written informed consent was obtained from each patient before the research.

## Conflicts of Interest

The authors declare no conflicts of interest.

## Supporting information


**Figure S1.** Analysis of differences in the alpha and beta diversity of gut microbiome between the control and HCC groups. (A) Alpha diversity between the two groups is described by the Shannon index. (B) Alpha diversity between the two groups described by the Simpson index. (C) Beta diversity between the two groups described by PCoA of weighted Unifrac distance matrix. Abbreviations: HCC, hepatocellular carcinoma; PCoA, principal coordinated analysis.

**Figure S2.** Results of the PICRUSt2 analysis at KEGG pathway.

**Figure S3.** Results of gut microbe‐based machine learning models by LASSO regression analysis. (A) Lasso regularization path. (B) Lasso coefficient path.

**Table S1.** Comparison of essential clinical characteristics before and after PSM in patients with and without hepatocellular carcinoma.
**Table S2.** Univariate and multivariable logistic regression analysis of clinical factors associated with hepatocellular carcinoma.

## Data Availability

The data included in the study are available from the corresponding author upon reasonable request.
