# Supplementary figures and images for: Gut Microbiota as Mediator and Moderator Between Hepatitis B Virus and Hepatocellular Carcinoma: A Prospective Study
Source: Cancer Med. 2024 Dec 19;13(24):e70454. doi: 10.1002/cam4.70454 (PMC11659115; doi:10.1002/cam4.70454)

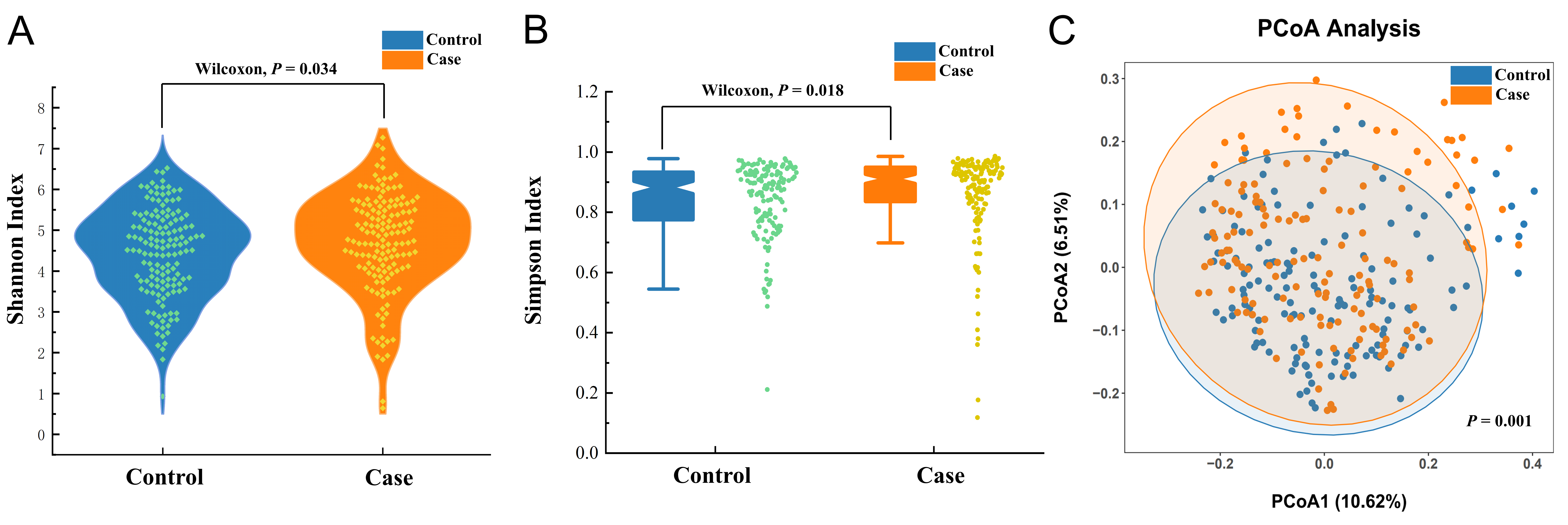

Supplement: Supplementary file 1 — Figure S1. Analysis of differences in the alpha and beta diversity of gut microbiome between the control and HCC groups. (A) Alpha diversity between the two groups is described by the Shannon index. (B) Alpha diversity between the two groups described by the Simpson index. (C) Beta diversity between the two groups described by PCoA of weighted Unifrac distance matrix. Abbreviations: HCC, hepatocellular carcinoma; PCoA, principal coordinated analysis. [file CAM4-13-e70454-s002.tif]

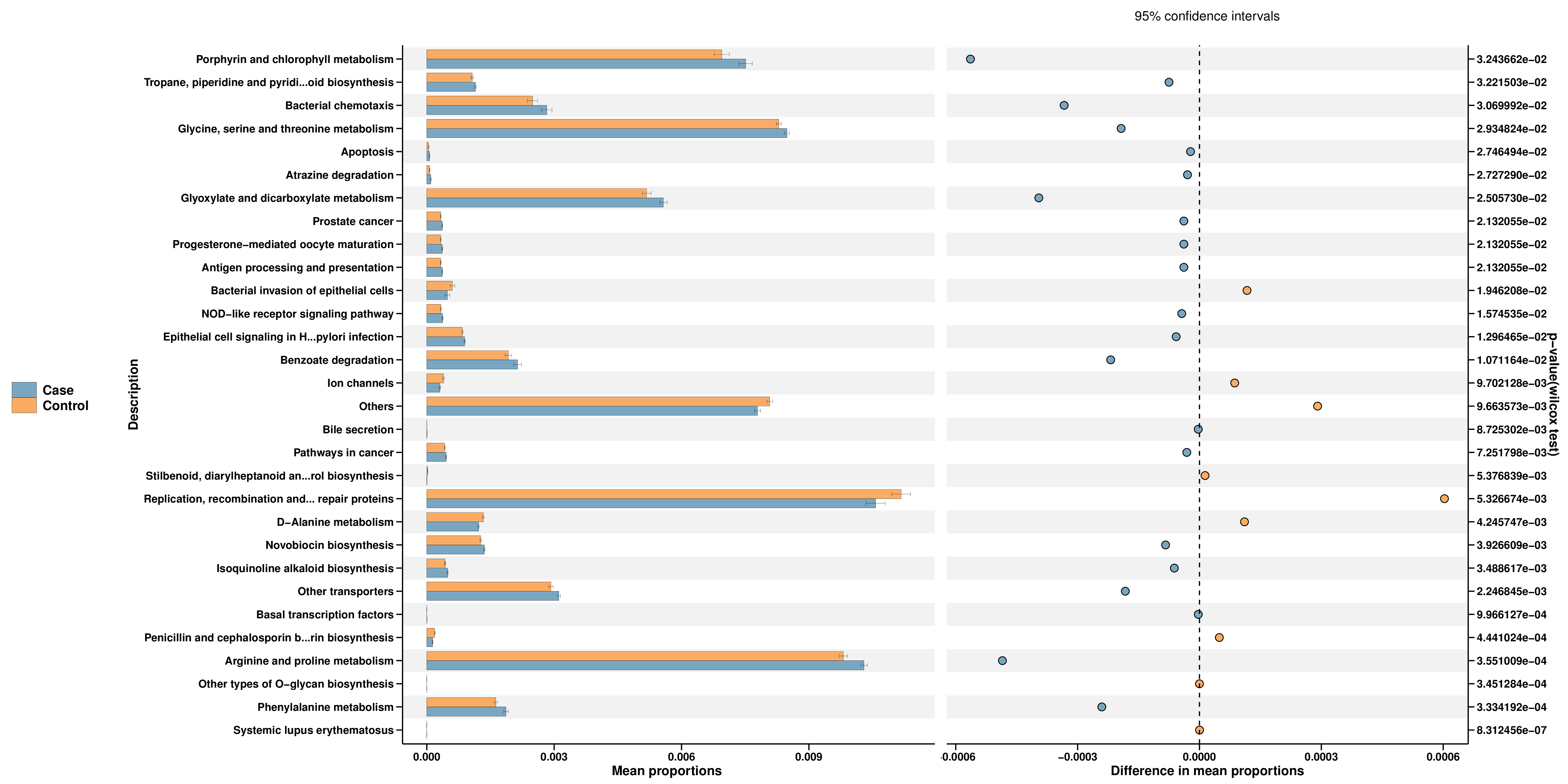

Supplement: Supplementary file 2 — Figure S2. Results of the PICRUSt2 analysis at KEGG pathway. [file CAM4-13-e70454-s003.tif]

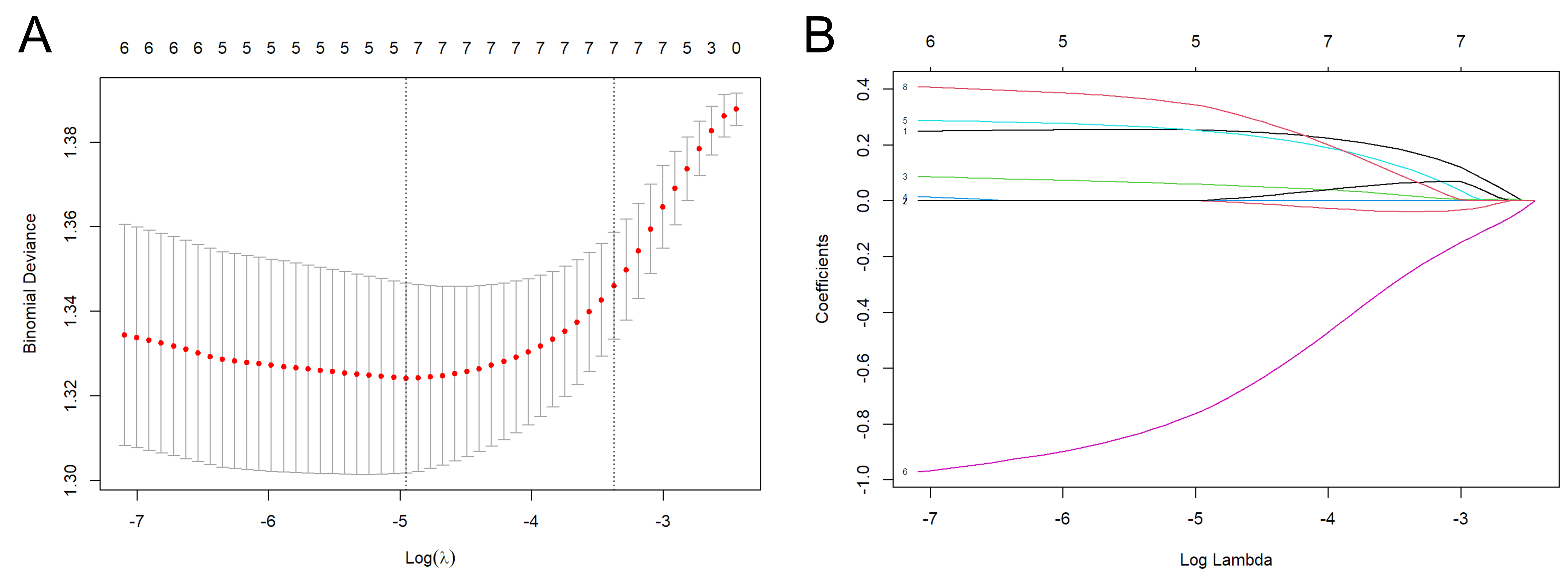

Supplement: Supplementary file 3 — Figure S3. Results of gut microbe‐based machine learning models by LASSO regression analysis. (A) Lasso regularization path. (B) Lasso coefficient path. [file CAM4-13-e70454-s004.tif]
