# Supplementary material for: Gut Microbiota as Mediator and Moderator Between Hepatitis B Virus and Hepatocellular Carcinoma: A Prospective Study
Source: Cancer Med. 2024 Dec 19;13(24):e70454. doi: 10.1002/cam4.70454 (PMC11659115; doi:10.1002/cam4.70454)
Supplement: Supplementary file 4 — Table S1. Comparison of essential clinical characteristics before and after PSM in patients with and without hepatocellular carcinoma. Table S2. Univariate and multivariable logistic regression analysis of clinical factors associated with hepatocellular carcinoma. [file CAM4-13-e70454-s001.docx]

**Supplementary Table S1.** Comparison of essential clinical characteristics before and after PSM in patients with and without hepatocellular carcinoma

| **Characteristics** | **Before PSM** | | | **After PSM** | | |
| --- | --- | --- | --- | --- | --- | --- |
|  | **Non-HCC** | **HCC** | ***P*-value** | **Non-HCC** | **HCC** | ***P*-value** |
|  | **(N=197)** | **(N=374)** |  | **(N=147)** | **(N=147)** |  |
| Age(year), mean ± SD | 55.29 ± 12.10 | 59.02 ± 11.20 | < 0.001 | 57.18 ± 12.08 | 59.56 ± 16.02 | 0.153 |
| Gender, n (%) |  |  | < 0.001 |  |  | 1.000 |
| Female | 102 (51.80) | 52 (13.90) |  | 53 (36.10) | 52 (35.40) |  |
| Male | 95 (48.20) | 322 (86.10) |  | 94 (63.90) | 95 (64.60) |  |
| BMI (kg/m^2^), mean ± SD | 23.42 ± 3.15 | 23.17 ± 3.02 | 0.364 | 23.37 ± 3.05 | 23.08 ± 3.23 | 0.437 |
| Diet, n (%) |  |  | 0.719 |  |  | 0.656 |
| Meat Diet | 17 (8.60) | 37 (9.90) |  | 15 (10.20) | 12 (8.20) |  |
| Vegetarian Diet | 16 (8.10) | 36 (9.60) |  | 14 (9.50) | 11 (7.50) |  |
| Balanced Diet | 164 (83.20) | 301 (80.50) |  | 118 (80.30) | 124 (84.40) |  |
| Smoking, n (%) |  |  | < 0.001 |  |  | 0.440 |
| No | 151 (76.60) | 220 (58.80) |  | 101 (68.70) | 108 (73.50) |  |
| Yes | 46 (23.40) | 154 (41.20) |  | 46 (31.30) | 39 (26.50) |  |
| Alcohol, n (%) |  |  | < 0.001 |  |  | 0.293 |
| No | 152 (77.20) | 230 (61.50) |  | 103 (70.10) | 112 (76.20) |  |
| Yes | 45 (22.80) | 144 (38.50) |  | 44 (29.90) | 35 (23.80) |  |
| Hepatitis B, n (%) |  |  | < 0.001 |  |  | < 0.001 |
| No | 145 (73.60) | 72 (19.30) |  | 102 (69.40) | 28 (19.00) |  |
| Yes | 52 (26.40) | 302 (80.70) |  | 45 (30.60) | 119 (81.00) |  |
| Cirrhosis, N (%) |  |  | 0.050 |  |  | 0.702 |
| No | 150 (76.10) | 254 (67.90) |  | 105 (71.40) | 101 (68.70) |  |
| Yes | 47 (23.90) | 120 (32.10) |  | 42 (28.60) | 46 (31.30) |  |
| Type 2 diabetes, n (%) |  |  | 0.509 |  |  | 0.225 |
| No | 162 (82.20) | 317 (84.80) |  | 116 (78.90) | 125 (85.00) |  |
| Yes | 35 (17.80) | 57 (15.20) |  | 31 (21.10) | 22 (15.00) |  |
| Hypertension, n (%) |  |  | 0.747 |  |  | 0.702 |
| No | 132 (67.00) | 257 (68.70) |  | 105 (71.40) | 101 (68.70) |  |
| Yes | 65 (33.00) | 117 (31.30) |  | 42 (28.60) | 46 (31.30) |  |

Abbreviations: HCC, hepatocellular carcinoma; PSM, propensity score matching; BMI, body mass index; SD, standard deviation

**Supplementary Table S2.** Univariate and multivariable logistic regression analysis of clinical factors associated with hepatocellular carcinoma.

| **Characteristics** | **Non-HCC** | **HCC** | **Univariate logistic analysis** | | **Multivariate logistic analysis** | |
| --- | --- | --- | --- | --- | --- | --- |
|  | **(N=147)** | **(N=147)** | **OR (95% CI)** | ***P*-value** | **OR (95% CI)** | ***P*-value** |
| Age (year), mean ± SD | 57.18 ± 12.08 | 59.56 ± 16.02 | 1.012 (0.996-1.028) | 0.153 |  |  |
| Gender, n (%) |  |  | 1.030 (0.639-1.660) | 1.000 |  |  |
| Female | 53 (36.10) | 52 (35.40) |  |  |  |  |
| Male | 94 (63.90) | 95 (64.60) |  |  |  |  |
| BMI (kg/m^2^), mean ± SD | 23.37 ± 3.05 | 23.08 ± 3.23 | 0.971 (0.903-1.045) | 0.437 |  |  |
| Hepatitis B, n (%) |  |  | 9.633 (5.609-16.545) | <0.001 | 11.287 (5.554-24.318) | <0.001 |
| No | 102 (69.40) | 28 (19.00) |  |  |  |  |
| Yes | 45 (30.60) | 119 (81.00) |  |  |  |  |
| Smoking, n (%) |  |  | 1.179 (1.018-1.315) | 0.440 |  |  |
| No | 101 (68.70) | 108 (73.50) |  |  |  |  |
| Yes | 46 (31.30) | 39 (26.50) |  |  |  |  |
| Alcohol drinking, n (%) |  |  | 1.732 (1.436-1.928) | 0.293 |  |  |
| No | 103 (70.10) | 112 (76.20) |  |  |  |  |
| Yes | 44 (29.90) | 35 (23.80) |  |  |  |  |
| Cirrhosis, n (%) |  |  | 1.139 (0.691-1.877) | 0.702 |  |  |
| No | 105 (71.40) | 101 (68.70) |  |  |  |  |
| Yes | 42 (28.60) | 46 (31.30) |  |  |  |  |
| AFP (ng/mL), n (%) |  |  | 6.862 (4.315-8.482) | <0.001 | 7.922 (5.074-9.908) | <0.001 |
| ≤200 | 146 (99.30) | 100 (68.00) |  |  |  |  |
| >200 | 1 (0.70) | 47 (32.00) |  |  |  |  |
| CEA (ng/mL), n (%) |  |  | 1.000 (0.447-2.237) | 1.000 |  |  |
| ≤5 | 134 (91.20) | 134 (91.20) |  |  |  |  |
| >5 | 13 (8.80) | 13 (8.80) |  |  |  |  |
| CA199 (ng/mL), n (%) |  |  | 1.050 (0.569-1.949) | 1.000 |  |  |
| ≤35 | 123 (83.70) | 122 (83.00) |  |  |  |  |
| >35 | 24 (16.30) | 25 (17.00) |  |  |  |  |
| TBIL (μmol/L), median (IQR) | 14.00 (9.50, 22.00) | 11.00 (8.00, 17.00) | 0.987 (0.975-0.999) | 0.023 | 0.992 (0.968-1.025) | 0.578 |
| DBIL (μmol/L), median (IQR) | 5.00 (3.00, 10.50) | 5.00 (3.00, 7.00) | 0.968 (0.945-0.992) | 0.002 | 1.002 (0.906-1.061) | 0.955 |
| Albumin (g/L), mean ± SD | 38.64 ± 6.26 | 37.96 ± 4.81 | 0.978 (0.939-1.019) | 0.297 |  |  |
| Albumin / Globulin, mean ± SD | 1.28 ± 0.32 | 1.23 ± 0.27 | 0.560 (0.257-1.217) | 0.143 |  |  |
| ALT (μmol/L), median (IQR) | 27.00 (16.50, 49.00) | 26.00 (19.00, 37.50) | 0.992 (0.986-0.998) | 0.003 | 0.989 (0.978-0.997) | 0.038 |
| AST (μmol/L), median (IQR) | 28.00 (21.00, 51.00) | 34.00 (27.00, 48.50) | 0.998 (0.993-1.003) | 0.427 |  |  |
| Total Cholesterol (mmol/L), mean ± SD | 4.68 ± 1.17 | 4.50 ± 1.10 | 0.867 (0.707-1.063) | 0.170 |  |  |
| Triglyceride (mmol/L), median (IQR) | 1.40 (0.90, 1.90) | 1.10 (0.80, 1.40) | 0.480 (0.33-0.698) | <0.001 | 0.470 (0.271-0.763) | 0.005 |
| Child-Pugh, n (%) |  |  | 1.962 (1.033-3.729) | 0.001 | 2.168 (1.039-3.626) | 0.011 |
| A | 121 (82.30) | 140 (95.20) |  |  |  |  |
| B | 26 (17.70) | 7 (4.80) |  |  |  |  |
| PT (s), mean ± SD | 13.75 ± 1.55 | 14.10 ± 1.42 | 1.179 (1.004-1.385) | 0.042 | 1.053 (0.827-1.356) | 0.682 |
| Fibrinogen (g/L), median (IQR) | 2.80 (2.40, 3.80) | 3.00 (2.40, 3.60) | 0.947 (0.797-1.125) | 0.534 |  |  |
| WBC (10^9^/L), median (IQR) | 5.70 (4.40, 7.40) | 5.30 (4.20, 6.70) | 0.906 (0.815-1.007) | 0.064 | 1.036 (0.891-1.204) | 0.643 |
| NEU (10^9^/L), median (IQR) | 3.40 (2.60, 4.60) | 3.40 (2.40, 4.30) | 0.920 (0.815-1.038) | 0.172 |  |  |
| Monocytes (10^9^/L), median (IQR) | 0.40 (0.30, 0.60) | 0.40 (0.30, 0.60) | 1.960 (1.689-5.58) | 0.206 |  |  |
| Lymphocyte (10^9^/L), median (IQR) | 1.50 (1.00, 1.90) | 1.40 (1.00, 1.80) | 0.734 (0.496-1.084) | 0.119 |  |  |
| Hemoglobin (g/L), median (IQR) | 129.00 (117.00, 144.00) | 130.00 (117.00, 145.00) | 1.007 (0.996-1.019) | 0.221 |  |  |
| Platelet (10^9^/L), median (IQR) | 192.00 (139.50, 241.50) | 167.00 (117.00, 218.50) | 0.998 (0.995-1.001) | 0.084 | 1.003 (0.999-1.007) | 0.196 |

Abbreviations: HCC, hepatocellular carcinoma; BMI, body mass index; SD, standard deviation; AFP, alpha-fetoprotein; CEA, carcinoembryonic antigen; CA199, carbohydrateatigen19-9; TBIL, total bilirubin; DBIL, direct bilirubin; ALT, alanine transaminase; AST, aspartate transaminase; PT, prothrombin time; IQR, interquartile range; WBC, White Blood Cell; NEU, neutrophilic granulocyte
